# Voluntary Medical Associations

**Published:** 1847-03

**Authors:** 


*Voluntary Medical Associations.*—It will be perceived by the circular, which we have transferred to our columns, emanating from a committee of the State Society, that resolutions were adopted at the meeting of the Society recommending the formation of voluntary associations similar to the Academy of Medicine recently instituted at the city of New-York. As regards Buffalo, the profession here have already moved in this matter, having organized an association, with great unanimity of feeling, which is now in successful operation, meetings for reports, discussions, &c., being held monthly. Our views of the importance of this subject we have heretofore taken occasion to express at some length. We regard it as of prime and essential importance in its relations to the cultivation of Medical science, the diffusion of scientific information, and the elevation of the character of the Medical profession. In all cities and villages containing half a dozen respectable physicians, much good will result from the formation of associations for mental improvement, and professional and social intercourse. To be convinced of the value of such intercourse, let every physician inquire of himself,if,in meeting with an intelligent professional friend, and conversing with him casually on medical subjects for a single hour, he does not generally carry away some facts, or hints, for useful reflection, or practical application. This has been our experience, and we presume it is that of others. The benefit in such cases is recipocal; each receives and imparts some valuable ideas. And it is simply a greater extension of this advantage, for the several members of the profession residing in the same place, or neighborhood, to hold frequent meetings, each actuated with a desire to contribute his quota of information, and to avail himself of the accumulated fund contributed by his associates.

But in addition to the professional improvement which each member of such an association derives, the influences exerted upon the profession are of a most desirable character. By associating together, and exchanging views upon subjects of common interest, community of feeling is engendered; personal jealousies and animosities are prevented; and the practice of medicine is not only rendered more agreeable, but its respectability in the eyes of the community is enhanced. The disagreements and quarrels of medical men, which have long since passed into a proverb, have, doubtless, been greatly exaggerated; yet, candour compels the acknowledgement that there is too often an unfortunate deficiency in that spirit of mutual consideration, and friendliness, which should characterise the professors of a pursuit, the essence of which is philanthropy, Why should not physicians seem to each other what they appear to others, warm hearted, disinterested generous men—for these we may claim as the qualities which belong to them as a body? It is because they occupy with regard to each other, too often isolated positions: they do not know each other, and mutual ignorance of motives begets misunderstandings and distrust. How many have lived in due contiguity for years entertaining feelings of suspicion or animosity, when a better acquaintance would have cemented friendships from which the greatest comforts and and profit might have been derived!

We do not. however, of course, contend that every member of the Medical Profession is always actuated solely by principles of honor and integrity. Like every other class, is made up of the good, the bad, and indifferent. Some will act justly and fairly upon compulsion only. Here, too, the benefits of associations are apparent, claiming, as we may with propriety, that the majority are disposed to act justly, and for the best interests of the profession. They have the power to enforce the observanee of such rules of conduct as the honor and usefulness of the profession shall seem to require. No member of a liberal profession canlong persist with propriety, in a course of dishonest, or dishonorable practices, with the continued voice of his brethren against him. Sooner or later he will discover that he is warring against his own interests; if he sees fit to continue in evil doing, the profession is enabled, by concert of action, to preserve its character by repudiating his direlection from duty and disclaiming all fellowship with the offender.

The position of the Medical Profession in this country, is such, that reform and progress are to be effected chiefly by the profession alone. Much as the public interest are concerned in the matter, it is not to be expected that the public, by legislation or otherwise, will co-operate to much extent in the furtherance of objects which relate to the advancement of Medical Service or the character of the profession. It is for members of the profession to make the profession what it is to become, as it has become already what it is through their instrumentality. There is no alternative but self-reliance; and impressed with this conviction it is for the profession to unite in effecting all that can be done for the interests of a pursuit which every true member of the profession must love and venerate. To accomplish any thing worthy of our pursuit, and of ourselves, we must come together, and make available all the advantages of associations. It affords gratifying evidence that a spirit of greater activity and zeal is beginning to pervade the ranks of the profession, that new societies are being formed, and that new life is infused into those already instituted. The organization of the Academy of Medicine in the city of New-York, with such unanimity and enthusiasm, is a sign of happy import. The action of our State society which has suggested these remarks, is of good augury; and we are pleased to learn that more interest was exhibited among the members present at its late session than has been usual on such occasions. The cordial concurrence in the plan of a national convention which we perceive is manifested in all sections of our country, also,affords little room to doubt that the general experiment of forming a national association representing the profession of the whole county will be entirely successful. We fervently trust that this growing ardor will not abate, but increase and expand, until it reaches every district and individual. Nothing will tend so much to diffuse it as the habit of frequently convening for purposes already mentioned. This is absolutely indispensable in order to devolope and nourish that *esprit du corps* which will unite the profession in a phalanx whose onward course in the march of improvement will realize its capabilities for progress, and conform to the spirit of the age. We conceive that they who feel imbued with a deep concern for the progress of Medical Science, and the Medical profession, can in no mode render more efficient aid to the cause which they have at heart, than by promoting, in every way, the formation of associations and the attainment of their legitimate objects.

